# Using accelerometers to develop time-energy budgets of wild fur seals from captive surrogates

**DOI:** 10.7717/peerj.5814

**Published:** 2018-10-26

**Authors:** Monique A. Ladds, Marcus Salton, David P. Hocking, Rebecca R. McIntosh, Adam P. Thompson, David J. Slip, Robert G. Harcourt

**Affiliations:** 1 School of Mathematics and Statistics, Victoria University of Wellington, Wellington, New Zealand; 2 Marine Predator Research Group, Macquarie University, Sydney, New South Wales, Australia; 3 School of Biological Sciences, Monash University, Melbourne, Victoria, Australia; 4 Research Department, Phillip Island Nature Parks, Phillip Island, Victoria, Australia; 5 TAL Life Limited, Melbourne, Victoria, Australia; 6 Taronga Conservation Society Australia, Sydney, New South Wales, Australia

**Keywords:** Accelerometer, Otariid, Activity budget, Time-energy budget, Fitness, Daily energy expenditure (DEE), Machine learning

## Abstract

**Background:**

Accurate time-energy budgets summarise an animal’s energy expenditure in a given environment, and are potentially a sensitive indicator of how an animal responds to changing resources. Deriving accurate time-energy budgets requires an estimate of time spent in different activities and of the energetic cost of that activity. Bio-loggers (e.g., accelerometers) may provide a solution for monitoring animals such as fur seals that make long-duration foraging trips. Using low resolution to record behaviour may aid in the transmission of data, negating the need to recover the device.

**Methods:**

This study used controlled captive experiments and previous energetic research to derive time-energy budgets of juvenile Australian fur seals (*Arctocephalus pusillus)* equipped with tri-axial accelerometers. First, captive fur seals and sea lions were equipped with accelerometers recording at high (20 Hz) and low (1 Hz) resolutions, and their behaviour recorded. Using this data, machine learning models were trained to recognise four states—foraging, grooming, travelling and resting. Next, the energetic cost of each behaviour, as a function of location (land or water), season and digestive state (pre- or post-prandial) was estimated. Then, diving and movement data were collected from nine wild juvenile fur seals wearing accelerometers recording at high- and low- resolutions. Models developed from captive seals were applied to accelerometry data from wild juvenile Australian fur seals and, finally, their time-energy budgets were reconstructed.

**Results:**

Behaviour classification models built with low resolution (1 Hz) data correctly classified captive seal behaviours with very high accuracy (up to 90%) and recorded without interruption. Therefore, time-energy budgets of wild fur seals were constructed with these data. The reconstructed time-energy budgets revealed that juvenile fur seals expended the same amount of energy as adults of similar species. No significant differences in daily energy expenditure (DEE) were found across sex or season (winter or summer), but fur seals rested more when their energy expenditure was expected to be higher. Juvenile fur seals used behavioural compensatory techniques to conserve energy during activities that were expected to have high energetic outputs (such as diving).

**Discussion:**

As low resolution accelerometry (1 Hz) was able to classify behaviour with very high accuracy, future studies may be able to transmit more data at a lower rate, reducing the need for tag recovery. Reconstructed time-energy budgets demonstrated that juvenile fur seals appear to expend the same amount of energy as their adult counterparts. Through pairing estimates of energy expenditure with behaviour this study demonstrates the potential to understand how fur seals expend energy, and where and how behavioural compensations are made to retain constant energy expenditure over a short (dive) and long (season) period.

## Introduction

An animal’s fitness can be assessed by its ability to survive and reproduce in a given environment (Orr, 2009). Time-energy budgets are a useful measure of one aspect of animal fitness, as they describe the energy spent and energy gained over a specific period in an animal’s life (Boyd, 2002). Animals gain energy by eating and metabolising food, and expend energy largely through basal metabolic rate (BMR), digestion, thermoregulation and activity, with excess energy available for growth and reproduction (Costa & Williams, 1999). Time-energy budgets that quantify both the time animals spend engaged in different activities and the energetic costs associated with those activities can be used to determine whether animals are in positive energy balance (Travis, 1982).

Air-breathing marine mammals, such as fur seals, that forage on aquatic prey have challenging constraints when acquiring energy. They dive repeatedly, may travel long distances to foraging sites (an energetically intensive strategy), and must return to the surface to breathe, only diving for as long as their oxygen stores allow (Gerlinsky, Trites & Rosen, 2014). As relatively small marine mammals, fur seal thermoregulatory costs at sea are high compared to terrestrial counterparts, because water conducts heat 25 times faster than air (Hind & Gurney, 1997) and the heat increment of feeding (HIF) also consumes energy (Rosen & Trites, 1997). However, thermoregulation and HIF are negligible in comparison to resting metabolic rate and activity that contribute to the largest variation in energy expenditure (Dalton, Rosen & Trites, 2015). How fur seals acquire and allocate energy to key processes may be understood through constructing time-energy budgets developed by calculating daily energy expenditure (DEE) and resolved by recording the duration of various activities and multiplying these by their associated energetic cost (Goldstein, 1988).

Calculating the costs associated with different activities from wild fur seals is difficult and often expensive, but estimates of the energetic costs associated with different activities have been made from laboratory experiments using captive surrogates and respirometry (Ladds, Slip & Harcourt, 2016). While respirometry measures metabolic rate (and thus energy expenditure) accurately, its field applications are limited (Halsey, 2011). If a proxy of the metabolic costs incurred by different activities can be developed using animal-borne sensors that identify these activities, such as accelerometers, then we can potentially measure activity-specific field metabolic rates as well (Cooke et al., 2014). But as the energetic outputs will vary for animal size, age and the time of year, this technology must be validated (Nathan et al., 2012).

Accelerometers have been used to define the behavioural state of a range of animals, validated through captive experiments (Diosdado et al., 2015; Wang et al., 2015). They can measure specific events, such as prey-capture (Volpov et al., 2015), identify a range of behaviours (Whitney et al., 2010) and define movement patterns (Shepard et al., 2008b). Dynamic body acceleration (DBA) or stroke rate, measured from accelerometers was promoted as a way to directly estimate energy expenditure in wild fur seals (Jeanniard-du Dot et al., 2016). However, this approach has recently been shown to be flawed, as the apparent relationship between DBA and energy expenditure is in fact time correlated with time, as both the independent variable (energy) and dependent variable (DBA or strokes) are both summed, thus introducing time into both sides of the equation (Halsey, 2017; Ladds et al., 2017a). Thus, a new way of estimating energy expenditure is needed.

Accelerometers can record at high resolution (multiple samples per second), to give a detailed picture of behaviour, but processing this amount of data post-collection is time-consuming and the amount of data collected may limit opportunities for uploading data remotely (Nathan et al., 2012). In addition, battery and memory limitations mean that if animals are at sea for long periods the period that can be sampled at a high resolution maybe relatively short compared to the total trip due to logger memory constraints (Halsey et al., 2009). Limiting the amount of accelerometry data that needs to be collected allows for smaller devices to be deployed, or for additional data from other sensors (such as temperature or orientation) to be collected. Fur seals make long-duration foraging trips over multiple days or weeks, and so analysing such trips maybe made simpler with low resolution (<10 Hz) recording.

In the pursuit of finding an appropriate and valid methodology of measuring wild fur seal energy expenditure and behaviour, the authors have conducted studies to investigate: the metabolic rate of fur seals during activity (Ladds, Slip & Harcourt, 2016); metabolic rates over seasons, sizes, sexes and species (Ladds, Slip & Harcourt, 2017); and how to classify behaviours from accelerometry (Ladds et al., 2017b; Ladds et al., 2016). What is missing now is a model connecting behaviour to its energetic cost and the application of the model to a wild population. To address this gap, we focus on the Australian fur seal (*Arctocephalus pusillus*). Australian fur seals are endemic to Australia, occupying much of the South-Eastern coast (Kirkwood & Goldsworthy, 2013). While much is known about adult females (Arnould & Hindell, 2001; Knox et al., 2014), few, if any, studies have focussed on juveniles.

This paper takes a five-step approach to achieving our goal. (1) Conduct behavioural experiments with captive seals and train machine learning models to automatically recognise four important behaviours (grooming, resting, travelling and foraging) with high (20 Hz) and low (1 Hz) resolution accelerometry. (2) Estimate the energetic cost of each behaviour based on previous research. (3) Collect accelerometer data from a sample of wild fur seals at high and low resolutions. (4) Apply the captive behaviour machine learning model to determine how much time is spent in each behaviour. (5) Apply the energetic cost of the behaviour in two locations (land and water) as a function of time and season to build an overall estimate of energy expenditure.

## Materials and Methods

### Behaviour validation experiments with captive fur seals and sea lions

To validate the use of accelerometers for classifying behaviours of wild fur seals we used captive surrogates at three Australian marine facilities; Dolphin Marine Magic Coffs Harbour, Underwater World Sunshine Coast, and Taronga Zoo Sydney, from August to November 2014 and in August 2015. We used two adult Australian fur seals (*Arctocephalus pusillus doriferus;* one male, one female), three New Zealand fur seals (*Arctocephalus forsteri;* two male adults, one male juvenile), one subantarctic fur seal (*Arctocephalus tropicalis;* juvenile male), and six Australian sea lions (*Neophoca cinerea;* two adult males, three adult females, one juvenile female). Fur seals had accelerometers attached with tape to the fur, while sea lions wore a custom fitted harness with the accelerometer sewn into a pocket (for details of the animals used see Ladds et al., 2017b, Table 1). We pooled data from all four species for training machine learning models because fur seals and sea lions (otariids) are conservative in morphology and including species and/or attachment method as a factor only improves the accuracy of such models by ∼5% (Ladds et al., 2017b). This study was conducted under permits from Macquarie University ethics committee (ARA-2012_064) and Taronga ethics committee (4c/10/13).

Tri-axial accelerometers (CEFAS Technology Ltd, Lowestoft, UK) were set to record −8 g to +8 g at 1 Hz and 25 Hz simultaneously, with a wet/dry sensor active, and behaviours typical of wild fur seals were video-recorded during training sessions. We observed two types of sessions; feeding and behaviour. The feeding sessions aimed to provide seals with large food items that required some form of processing prior to eating. Behaviour sessions also incorporated some feeding events with small fish that did not require processing. Fish were thrown in the pool so that seals had to “capture” them mid-water as they sank. These two behaviours constituted foraging. During each behaviour session seals were instructed to perform a series of natural behaviours from their known behavioural repertoire, such as porpoising, swimming and grooming. While seals could not reach depths they would achieve in the wild, their pools provided adequate space to perform behaviours typical of wild fur seals. Captive fur seals had access to both land and water during trials, similar to a wild fur seal near their haul-out. In addition, seals were trained to swim consistently below the water for several minutes to replicate a deep dive for another project. The experimental set-up and training allowed us to record behaviours that lasted from less than a second (grabbing fish from the water column) to several minutes (swimming or resting).

Behaviours were manually matched to the accelerometry by two investigators. Where behaviours recorded by the investigators did not match, they reviewed the video such that both reached agreement. Twenty-six behaviours were grouped into four behavioural categories—foraging, travelling, grooming and resting, in three locations—land, water surface and underwater (for details of the behaviours and their groups see Ladds et al., 2016, Table 2). Foraging behaviours consisted of searching for prey and prey handling limited to dead fish. Grooming was any behaviour used in body maintenance or thermoregulation. To thermoregulate at sea, fur seals float with either their hind flippers (jughandling) or their fore flippers (sailing) in the air. Grooming involves the use of flippers to scratch or rub the body, including the face to clean whiskers. Resting was any period of stillness, while travelling was any period involving movement that was not foraging or grooming (Ladds et al., 2017b).

### Estimate the energetic cost of each behaviour

#### Resting energy expenditure

Energy consumption when resting in water is related to water temperature for postabsorptive (not digesting) female and subadult Australian and New Zealand fur seals (Ladds, Slip & Harcourt, 2017). However, postprandial (digesting) resting metabolic rate (RMR) for pups of northern fur seal (*Callorhinus ursinus*) (Liwanag, 2010) and juvenile South American fur seals (*Arctocephalus australis*) (Dassis et al., 2014) is 1.6 times the postabsorptive rate and stays at this level for about 3.5 h. For simplicity, we assumed that fur seals were postabsorptive while on land, and postprandial in the water.

Resting in water:

(1) \begin{eqnarray*}{\mathrm{sSMR~ R}}_{\mathrm{water}} \left( \mathrm{l} {\mathrm{O}}_{2}~{\mathrm{kg}}^{-1} \right) =1.6(0.00195+0.00029 \left( \text{water temp}. \right) \left( \text{duration} \right) ).\end{eqnarray*}

Because no measure of RMR on land for juvenile Australian fur seals was available we used the mass specific standard metabolic rate (sSMR) of a subadult New Zealand fur seal in water (Ladds, Slip & Harcourt, 2017). As northern fur seal pups and southern sea lion subadult males both had ∼30% lower RMR on land than in water (Dassis et al., 2012; Donohue et al., 2000), this assumption was applied to our RMR estimation on land. In addition, to account for a seasonal effect on sSMR in New Zealand fur seals (Ladds, Slip & Harcourt, 2017), we calculated a summer and a winter energy consumption (Eqs. (2.1)–(2.2)).

Winter RMR on land:

(2.1) \begin{eqnarray*}{\mathrm{sSMR~ R}}_{\mathrm{winter,land}} \left( \mathrm{l} {\mathrm{O}}_{2} {\mathrm{kg}}^{-1} \right) =(0.007\times 0.7)(\text{duration}).\end{eqnarray*}

Summer RMR on land:

(2.2) \begin{eqnarray*}{\mathrm{sSMR~ R}}_{\mathrm{summer,land}} \left( \mathrm{l} {\mathrm{O}}_{2} {\mathrm{kg}}^{-1} \right) =(0.009\times 0.7)(\text{duration}).\end{eqnarray*}

#### Active energy expenditure from foraging and travelling

We combined foraging and travelling as, despite having many studies of the energetic cost of diving in seals e.g., (Rosen et al., 2016; Williams et al., 2004), there is yet to be a study evaluating the cost of travelling at the surface. The time an animal spent active (foraging and travelling) was multiplied by the average energy expenditure estimated in Ladds, Slip & Harcourt (2016). In their study seals swam below the surface stroking constantly, thus the energetic cost of activity was estimated (as opposed to foraging or travelling per se) (Ladds, Slip & Harcourt, 2016; Rosen et al., 2016; Williams et al., 2004).

There have been no estimates of the cost of travelling on land for any pinniped, though experimentally the cost of movement on land is probably much greater than in water. In semi-aquatic water rats (*Hydromys chrysogaster*) the metabolic cost of running was around 13–40% more than swimming when moving at equal speeds, and for platypus (*Ornithorhynchus anatinus*), the cost of walking was 2.1 times the cost of swimming (Fish et al., 2001). As terrestrial locomotion in otariids is more similar to platypus than water rat, we assumed that the cost of movement on land is twice that in water. As activity compensates for some of the additional costs of cold water (Liwanag et al., 2009) we assumed that the energy expenditure for winter and summer was the same (Eq. (3.1)).

Energy expended from activity (foraging and travelling):

(3.1) \begin{eqnarray*}{A}_{\mathrm{water,winter/ summer}} \left( \mathrm{l} {\mathrm{O}}_{2} {\mathrm{kg}}^{-1} \right) & =0.0303(\text{duration})\end{eqnarray*}

(3.2) \begin{eqnarray*}{A}_{\mathrm{land,winter/ summer}} \left( \mathrm{l} {\mathrm{O}}_{2} {\mathrm{kg}}^{-1} \right) & =2(0.0303 \left( \text{duration} \right) ).\end{eqnarray*}

#### Grooming energy expenditure

The energy expended from grooming was estimated to be between 1.5 and 2 times the postprandial RMR and between 0.9 and 1.2 times postabsorptive RMR in northern fur seal pups depending on activity level (Liwanag, 2010). Considering our model generally only labelled active grooming, we assumed that grooming had an energetic cost twice that of in-water RMR (Eqs. (4.1)–(4.2)).

Energy expended from grooming in winter in water:

(4.1) \begin{eqnarray*}{\mathrm{G}}_{\mathrm{winter,water}} \left( \mathrm{l} {\mathrm{O}}_{2} {\mathrm{kg}}^{-1} \right) =(0.007\times 2)(\text{duration}).\end{eqnarray*}

Energy expended from grooming in summer in water:

(4.2) \begin{eqnarray*}{\mathrm{G}}_{\mathrm{summer,water}} \left( \mathrm{l} {\mathrm{O}}_{2} {\mathrm{kg}}^{-1} \right) =(0.009\times 2)(\text{duration}).\end{eqnarray*}

For grooming on land fur seals were assumed to be postabsorptive so they were assumed to have the same energetic output as resting or slightly higher.

### Wild fur seal data collection

We tracked juvenile Australian fur seals from two colonies, Seal Rocks (Phillip Island, Victoria Australia, 38° 52′S–145°11′E, *n* = 6) during austral winter of 2013 and Lady Julia Percy (Victoria, Australia, 38°52′S–142°00′E, *n* = 8) during austral summer of 2014. These sites are the largest breeding colonies for Australian fur seals, with each site containing approximately 25% of the total population of the species (Kirkwood et al., 2010). In Australian fur seals, suckling ceases after 1 year, puberty occurs in females at approximately 3 years old and in males at 4–5 years old (Arnould & Warneke, 2002). We used animals between one and three years of age that were independently foraging (i.e., juveniles) for this study.

**Figure 1:**
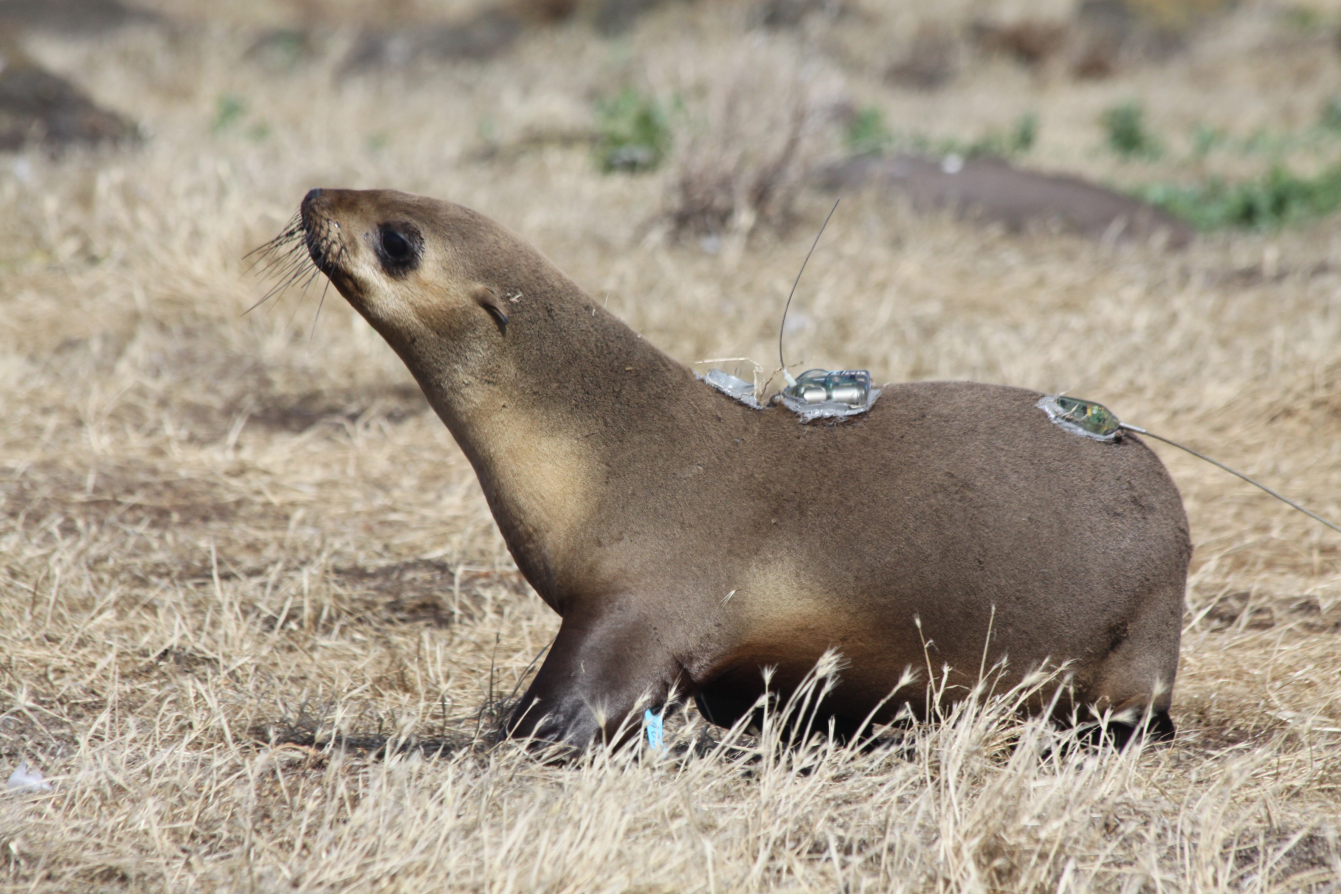
Juvenile Australian fur seal with three devices attached. Devices are CEFAS accelerometer, time depth recorder (TDR) and VHF. Source: DP Hocking.

We identified juveniles by their mature pelage (i.e., lacking the lanugo of pre-moult pups) and size (<1.5 m and 40 kg) and captured individuals using a modified hoop-net and isofluorane gas sedation (Gales & Mattlin, 1998), then measured standard length (straight-line), girth and mass. Numbered tags were applied to the trailing edge of both fore-flippers (Super Tags^®^, Dalton I.D. Systems Ltd, Henley-on-Thames, UK) to aid with identification and recapture. One of two types of location device (Kiwisat100, Sirtrack Ltd, New Zealand or Mk10; Wildlife Computers), a VHF transmitter (Sirtrack Ltd, 6 cm × 3  cm × 2 cm), and a tri-axial accelerometer G6A + (CEFAS technology Ltd, Lowestoft, UK) were glued directly to the fur on the dorsal midline of each fur seal (Fig. 1) using quick-setting epoxy (Araldite 2017; Aeropia Ltd, Crawley, UK or Araldite 268; Huntsman Advanced Materials, Victoria, Australia). A time-depth recorder (TDR) was also attached, either as part of the Mk10 device or a separate device (Mk9, Wildlife Computer; Fig. 1). The total instrument package mass (179 g–239 g) equated to <1% of the seal’s body mass and attached to maintain the lowest profile possible to minimise a drag effect. We observed animals until they had fully recovered from anaesthesia and released them at the site of capture. A minimum of 15 days lapsed before recapture (via hoop net and manual restraint), and devices were retrieved by cutting the hair beneath the glued instrument.

For the duration of the deployment, defined as from attachment until removal of a device, TDRs recorded depth (m) every second, and any drift in the depth sensors or error spikes were corrected prior to analyses using Zero-Offset Correction (Wildlife Computers, Redmond, WA, USA). Tri-axial accelerometers recorded acceleration on the *X*, *Y*, and *Z* axes at 1 sample per second (1 Hz) and temperature at 0.5 Hz. Accelerometers also recorded at a high resolution (20 Hz) when diving (depth > 1.5 m). This would sometimes continue after a diving event, giving high resolution data both at the surface and during diving.

To give an indication of time duration at sea, data from the TDRs were summarised into trips and dives. Trips started when a seal entered the water and ended when the seal hauled out, and excluded periods in water with minimal diving or dives <10 m (e.g., when seals rest at the water surface adjacent to colonies). Dives were defined as periods spent underwater below a minimum depth of 5 m to account for wave action at the surface. Fur seal physical parameters, trip duration, number of trips, average dive duration, mean maximum depth and maximum depth were derived from these parameters (Table S1).

### Predicting behaviours of wild fur seals from accelerometers

As the high resolution data were only recorded for wild seals while at sea, the high resolution captive data were subsampled to include only those behaviours that occurred in the water. Low resolution (1 Hz) data were recorded continuously for both the wild and captive seals. The wet/dry sensor of the accelerometers from the wild fur seals (hereafter wild data) was used to indicate when individuals were in water or on land to improve the predictability of the models. We tested the accuracy of both high and low resolution accelerometry to classify behaviours.

To determine the behavioural state of surrogates using accelerometers, gradient boosting models (GBM) were trained in R using the package ‘xgboost’ (Chen, He & Benesty, 2016). GBM models are an extension of a random forest, whereby they build a classification tree on a subset of the data, then use a subsequent tree to learn from the errors of the previous trees. Trees are built successively until a stopping criteria is met, and the trees built are averaged together to provide an estimate of classification (Friedman, 2002). Captive data were pooled and split into one of three epochs (the number of samples on which summary data are calculated) for training the GBM. For high resolution (20 Hz) data epochs of 13, 25 and 75 samples were tested, which correspond to 0.65, 1.25 and 3.75 s of data, respectively. For low resolution (1 Hz) data epochs of 7.0, 15.0 and 21.0 samples were tested, which corresponded to 7.0, 15.0 and 21.0 s of data respectively. Training and testing longer epochs was not possible because there were too few events lasted for longer than a few seconds, meaning there were not enough examples to train a model. Down-sampling (randomly selecting behaviours from a pool until a specified number is met) was used to ensure that the behaviour categories had an even number of samples (Ladds et al., 2017b).

We coded 52 summary statistics and added five covariates describing some characteristic of the individual or the event to the second stage of model testing. These were included as they have previously been demonstrated to make a small improvement on prediction performance of the models (Ladds et al., 2017b). The covariates included were device attachment method (harness or tape), age, mass, sex and species of the individual. We included where the behaviour occurred (surface, underwater or land) in all models. Location was determined first by the wet/dry switch which indicated whether a seal was in the water or on land, then once a seal was more than 1 m under the surface (as determined by the depth device) they were classified as underwater. Summary statistics calculated included: mean, median, standard deviation, skewness, kurtosis, minimum, maximum, absolute value, inverse covariance, autocorrelation trend (the coefficient derived from a linear regression) for each of the three axes. We also calculated *q* as the square-root of the sum-of-squares of the three axis (Nathan et al., 2012), and included pair-wise correlations of the three axes (*x*–*y*, *y*–*z*, *x*–*z*) (Ravi et al., 2005). The inclination as azimuth were calculated as per Nathan et al. (2012). We calculated three measures of dynamic body acceleration (DBA) by first using a running mean of each axis over 3 s to create a value for static acceleration. We then subtracted the static acceleration at each point from the raw acceleration value to create a value for partial dynamic body acceleration (PDBA). The values of PDBA on each axis were used to calculate overall dynamic body acceleration (ODBA) (Shepard et al., 2008a; Wilson et al., 2006) and vectorial dynamic body acceleration (VeDBA) (Qasem et al., 2012). The integral of the start and end point of ODBA and VeDBA for each epoch ODBA and VeDBA was calculated using the package “MESS” in R (Ekstrom, 2014, R Core Development Team, 2015).

GBM models were run with the full suite of summary statistics derived from captive data and run over a grid of parameters (for details see Table 2 and additional file 2 in Ladds et al., 2017b). The combination of parameters that resulted in the highest accuracies was chosen for implementation on the high and low resolution wild data. Within each epoch, wild data were categorized using predictions from the GBM model built with captive data that produced the highest cross-validation accuracy and kappa values. Accuracy is a measure of the proportion of true positives identified by the model, while kappa is a performance measure that accounts for investigators’ observations agreeing or disagreeing by chance. Behaviour events were categorised for the duration of each deployment. Events were considered different when either the location or the behaviour category changed for an epoch, and the change occurred for longer than 15 s. A sensitivity analysis was conducted on the probability of each event being assigned to a behaviour group. For wild data, each event was assigned a probability of it being each behaviour category and then classified as the behaviour that had the highest probability. To evaluate how well our models classified behaviours, behaviours that were selected with less than 80% chance of occurring were examined, and the behaviour with the next highest probability was extracted. This allowed us to see when the model may have ‘confused’ two categories.

### Apply the model to create time-energy budgets

**Figure 2:**
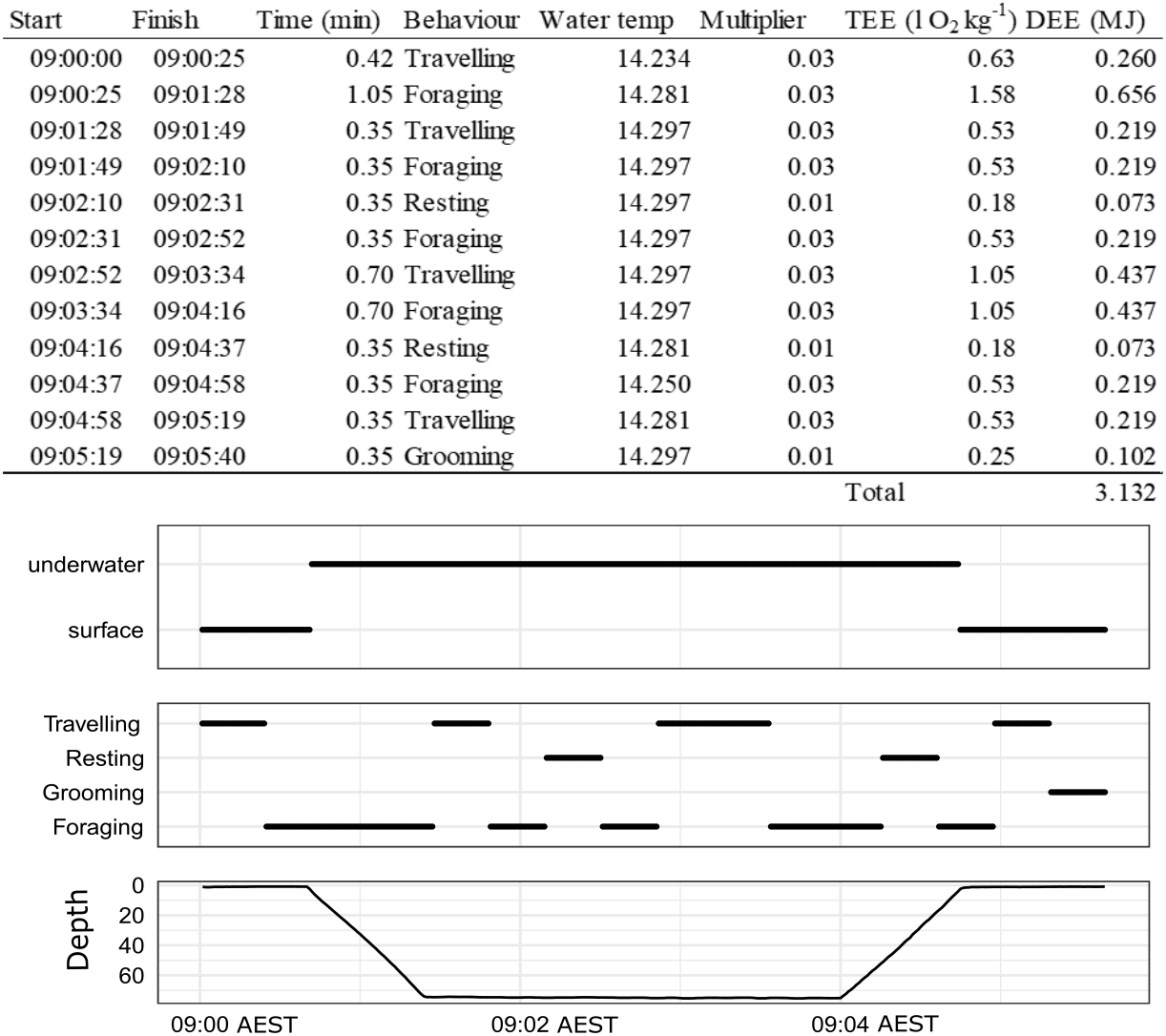
An example of how DEE is calculated for a single dive of a wild male juvenile Australian fur seal from Seal Rocks (winter), Victoria, Australia. Panels show location (underwater or surface), behaviour state (travelling, resting, grooming, foraging) and depth.

To build time-energy budgets, we estimated DEE (MJ) which was assumed to be a function of the energy expenditure (EE) of a given behaviour event, its duration, the season it occurred in and where it occurred (land or water) summed over 24 h periods (Table 1, Eq (5)). An example of how this is calculated over a single dive is given in Fig. 2. Details of the calculations and assumptions made for the energetic models are in Table 1. The overall energetics model is defined by the sum of all of the behaviour events (*e* = *e*…*E*) that occur in a 24 h period (from midnight to midnight) for *A* (activity), *G* (grooming) and *R* (resting) as a function of season *s* ( *s* = winter, summer) and location *l* ( *l* =  land, water):

(5) \begin{eqnarray*}\mathrm{DEE} \left( \mathrm{l} {\mathrm{O}}_{2} \right) =\sum _{e}^{E}{R}_{s,l}+\sum _{e}^{E}{A}_{s,l}+\sum _{e}^{E}{G}_{s,l}.\end{eqnarray*}

**Table 1:** Energy budget calculations and references for each behavioural and digestive state, accounting for location (land or water—where water includes surface and underwater) and season (winter or summer). All measures converted to l O_2_ kg^−1^ from original measure. Temperature in °C, duration in minutes, NZM3 is the reference to the seal used.

Energy expenditure	Digestive state	Location	Austral season	Energy expenditure equation (l O_2_ kg^−1^)	Reference
Resting	Postprandial	Water	Temp. related	S *R*_water_ = 1.6(0.002 + 0.0003 × water temp.)(duration)	Ladds, Slip & Harcourt, (2017, Fig 5D), Liwanag, (2010), Dassis et al., (2014)
Resting	Postabsorptive	Land	Winter	*R*_land,winter_ = 0.0049(duration)	Ladds, Slip & Harcourt (2017, Table 2 NFM3), Donohue et al. (2000), Dassis et al. (2014)
Resting	Postabsorptive	Land	Summer	*R*_land,summer_ = 0.0063(duration)	Ladds, Slip & Harcourt (2017, Table 2 NFM3), Liwanag (2010), Dassis et al. (2014)
Foraging/ travelling	N/A	Water	N/A	*A*_water_ = 0.0303(duration)	Ladds, Slip & Harcourt (2016, Table 1 NFM1)
Foraging/ travelling	N/A	Land	N/A	${A}_{\mathrm{land}}=0.0606 \left( \text{duration} \right) $	Ladds, Slip & Harcourt (2016, Table 1 NFM1), Fish et al., 2001
Grooming	Postprandial	Water	Winter	*G*_mwater,winter_ = 0.014(duration)	Ladds, Slip & Harcourt (2017, Table 2 NFM3), Liwanag (2010)
Grooming	Postprandial	Water	Summer	*G*_water,summer_ = 0.018(duration)	Ladds, Slip & Harcourt (2017, Table 2 NFM3), Liwanag (2010)
Grooming	Postabsorptive	Land	Winter	*G*_land,winter_ = 0.007(duration)	Ladds, Slip & Harcourt (2017, Table 2 NFM3), Donohue et al. (2000), Dassis et al. (2014)
Grooming	Postabsorptive	Land	Summer	*G*_land,summer_ = 0.009(duration)	Ladds, Slip & Harcourt (2017b, Table 2 NFM3), Liwanag (2010), Dassis et al. (2014)

See Table 1 for definitions and details of each behaviour state.

For reporting and comparison with other energetics papers, the total oxygen used was converted into MJ. First, the total energy expended was converted to kilocalories using a factor of 5 kcal per l O_2_, then converted to kilojoules using a conversion factor of 4.186  J  cal^−1^ (Williams et al., 2007).

### Statistical analysis

As it was not feasible to validate the captive model after applying it to the wild data, we conducted a sensitivity analysis on the probability of each event being assigned to a behaviour group. To do so, we adjusted the proportion of time wild fur seals spent in different behaviours (increasing some while decreasing others, such that the total proportion of time spent in the three behaviours always summed to 1) and investigated how it affected the overall DEE. We calculated 1,000 simulated proportions based on the actual range of time spent in each behavioural category, where each simulated proportion represented a day. We applied the energetics model to the simulated proportions and grouped the activities according to three behaviour categories: active (travelling and foraging) grooming and resting; then by two location categories: water and land. We then plotted the total DEE for the day against the simulated proportions for each of the five categories. As fur seals were of different sexes and were tagged in different seasons we tested for differences in DEE between sexes and seasons using *post-hoc* general linear hypothesis and a multiple comparison test via the Tukey method within the function *glht* from the package “multcomp” (Hothorn et al., 2013). Individual fur seal identification was included in models as a random factor and significance was set at *p* < 0.05. All analyses were completed in R (Version 3.1.3; R Core Development Team, 2015) and values reported as mean ± SEM. The datasets generated and analysed during the current study are available in the “Time-energy_budgets_from_accelerometers” repository: https://github.com/MoniqueLadds/Time-energy_budgets_from_accelerometers.git.

## Results

### Behaviour validation experiments with captive fur seals and sea lions

**Figure 3:**
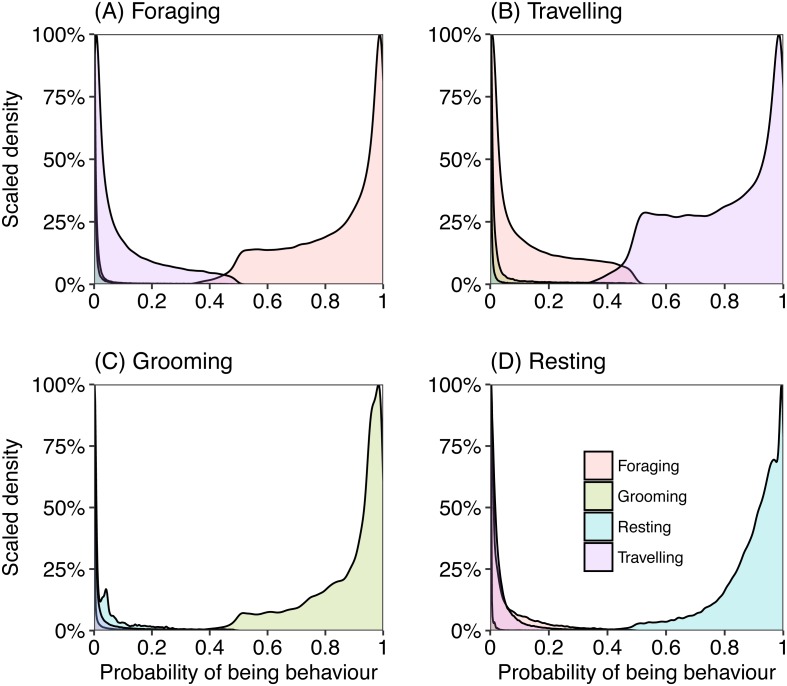
Density plots representing the probability of an epoch belonging to behavioural category (A) foraging, (B) travelling, (C) grooming and (D) resting, calculated from captive data. Each plot represents the probability of belonging to a behavioural category when the labelled category was predicted as the most likely class for that epoch.

Most epochs (99%) were assigned to a behaviour category with over 80% probability (Fig.  3). We investigated those behaviours that were assigned with a less than 80% (∼1% of total epochs) probability to understand where the model may have ‘confused’ behaviours. When the model was uncertain that an epoch was in the behaviour category “foraging” (less than chance), it generally predicted the epoch should be assigned as “travelling” and almost never “resting” or “grooming” (Fig. 3A). When there was uncertainty if an epoch should be categorised as “travelling”, with less than 50% chance, the behaviour category with the next highest probability was “foraging” (Fig. 3B). Grooming was rarely confused for other behaviours, but when there was uncertainty the model calculated “resting” with the next greatest probability (Fig. 3C). There was also little confusion with assigning an epoch to resting, but occasionally the model assigned a higher probability of foraging.

The models correctly classified surrogate behaviour (travelling, foraging, grooming, resting) with high accuracy (>68%), but the number of samples in an epoch used affected the results, where longer epochs (sampling time of behaviour) resulted in higher accuracies (Table 2). The best low resolution model (1 Hz) used 21 samples for an epoch, and the best high resolution model (20 Hz) used 75 samples for an epoch, both of which had the highest training, testing and kappa scores for their behaviour category. Given that the 1 Hz data classified behaviours with very high accuracy (90% out-of-sample using epochs of size 21, Table 2) and recorded for the duration of deployments on wild fur seals (20 Hz data only recorded while fur seals were diving and for a short time after), only the 1 Hz data for the activity budgets were analysed.

**Table 2:** Cross-validation (training) and out-of-sample (testing) accuracy for gradient boosting models (GBM) trained across a range of epochs using two datasets for all behaviours (ALL) and for behaviour in water (Water).

Behaviour	Hz	Epochs	Cross-validation accuracy	Out-of-sample accuracy	Kappa
ALL	1	7	78.3%	72.1%	71.1%
ALL	1	15	79.7%	86.8%	73.6%
ALL	1	21	80.8%	89.5%	73.6%
Water	20	13	63.2%	67.6%	68.6%
Water	20	25	72.4%	69.1%	63.2%
Water	20	75	82.7%	75.6%	76.9%

### Wild fur seal data collection

Three fur seals from Seal Rocks and six fur seals from Lady Julia Percy were successfully recaptured (recapture rates of 50% and 67% respectively) and accelerometer data obtained. Fur seals made between two and 45 trips with durations of between 30 min and nine days. Diving parameters (Table S1) were very similar between individuals from both sites. Fur seals in winter (from Seal Rocks) made fewer and longer foraging trips than fur seals in summer (from Lady Julia Percy). All other diving parameters were very similar between winter and summer fur seals (Table S1). Similarly, there were few differences in diving parameters for male and female juvenile fur seals.

### Predicting behaviours of wild fur seals from accelerometers

**Figure 4:**
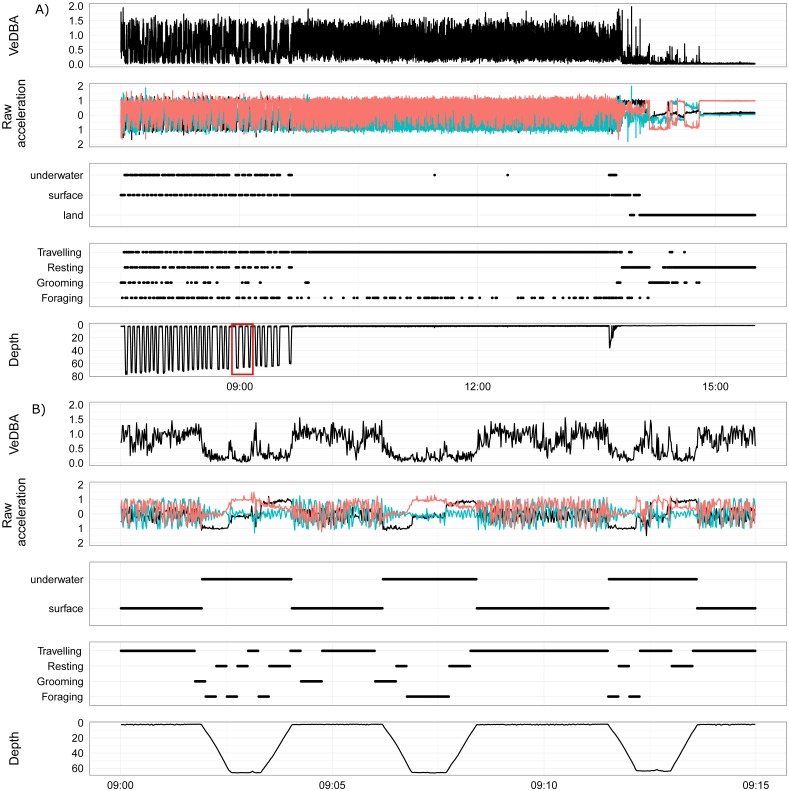
An example foraging bout, transition and haul-out of a female juvenile Australian fur seal from Lady Julia Percy, Victoria, Australia. (A, B) show VeDBA, raw acceleration of the *x*, *y* and *z* axis, location (underwater, surface or land), behaviour state (travelling, resting, grooming, foraging) and depth (time is in AEST). (A) Shows the end of a foraging bout, transiting back to land and then a short period of the haul out. The red box highlights the area of the dive that is displayed in (B). (B) shows three dives from the foraging bout.

Figure 4 is an example of the output produced by the activity model for a wild fur seal, showing the end of a foraging bout, travelling back to land and then a short period of resting on land (hauled out). This figure demonstrates the strengths and weaknesses of the GBM built from surrogates. The model was very good at predicting when the wild individual was resting, as there was very little movement in the accelerometer. But this feature resulted in the dive ascent also being classified as resting as the seal rose slowly through the water column with limited body movement (Fig. 4B). Grooming was also classified accurately; it predominantly occurred immediately prior to or following a dive, or during the first hour or so after hauling out. Foraging and travelling were frequently misclassified by the model (Fig. 3); most commonly, the descent of a dive was classified as foraging when it most likely should have been travelling, and foraging appeared periodically during long trips returning to the haul out site.

### Apply the model to create time-energy budgets

**Figure 5:**
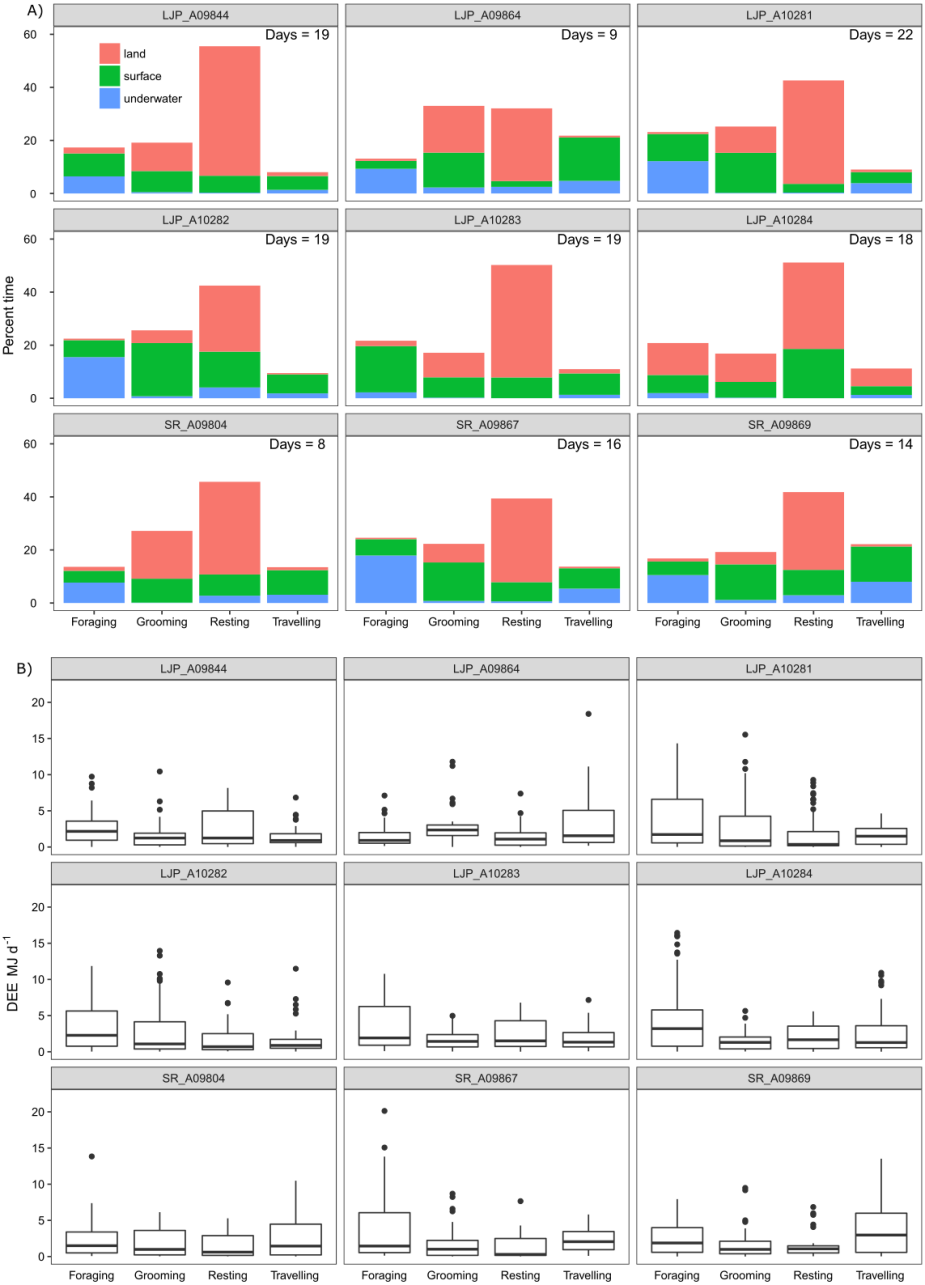
Activity (A) and energy (B) budgets for nine wild juvenile Australian fur seals—six deployed in summer and three deployed in winter. (A) Bars represent % of time spent in each type of activity over the duration of the deployment for the number of days presented in the top right-hand corner of plots. Colours represent the location of the behaviour. (B) Boxplots represent the minimum, 25% (Q1), median, 75% (Q3) and the upper limit (Q3 + 1.5 × the interquartile range (Q3 –Q1)) of DEE (MJ d^−1^) with outliers of the upper limit represented by points.

Overall, the time that fur seals spent in the three different locations was between 31–63% on land, between 3–25% underwater and between 28–47% at the surface of the water. The pressure sensor on the accelerometers on two of the wild fur seals (LJP_A10283 and LJP_A10284) failed for a portion of the deployment, which resulted in a significant underestimation of the time spent underwater. Each fur seal spent approximately half of their deployment resting (range 32–55%), predominantly on land (Fig. 5) and another 22% (range 17–33%) was used for grooming. Approximately 20% (range 13–25%) of fur seals’ time was foraging and approximately 12% travelling (range 8–22%).

There were no significant differences in the DEE for females (18.22 ± 5.91 MJ d^−1^) and males (18.86 ± 6.01 MJ d^−1^; *post-hoc* comparisons: *Z* =  − 0.35, *p* = 0.72) or for winter deployments (20.77 ± 7.00 MJ d^−1^) and summer (17.55 ± 5.30 MJ d^−1^; *post-hoc* comparisons: *Z* = 1.10, *p* = 0.27), which also represented site and year. Therefore, it was justifiable to pool the samples. The average DEE for wild individuals and locations pooled was 18.73 ± 5.73 MJ d^−1^ (range: 8.24–32.04 MJ d^−1^) and mass-specific DEE was 0.50 ±  0.14 MJ kg^−1^ d^−1^ (range: 0.08–0.81 MJ kg^−1^ d^−1^; Table 3). The maximum DEE was from a wild individual that spent 12 h continuously diving at sea (Fig. S1).

**Table 3:** Daily energy expenditure (DEE MJ d-1) for different behaviours on land, at the surface and underwater for nine juvenile Australian fur seals.

Behaviour	Average DEE (MJ d^−1^)	SD DEE	Max DEE	% total DEE	% Activity Budget
Land					
Resting	2.62	1.37	6.66	14.7%	35%
Grooming	1.05	0.86	3.77	5.9%	10%
Travelling^a^	1.22	1.52	7.69	6.9%	4%
Surface					
Resting	1.29	1.59	12.30	7.3%	9%
Grooming	1.59	1.46	7.80	8.9%	12%
Foraging	2.53	1.97	7.37	14.2%	8%
Travelling	2.46	2.38	12.24	13.8%	8%
Underwater					
Resting	0.27	0.41	2.15	1.5%	1%
Grooming	0.25	0.34	1.62	1.4%	2%
Foraging	3.30	3.46	17.41	18.5%	9%
Travelling	1.19	1.30	5.86	6.7%	3%
Total					
Resting	4.18	3.37	21.11	21.4%	45%
Grooming	2.89	2.65	13.20	14.8%	24%
Foraging	5.83	5.43	24.78	29.8%	16%
Travelling	6.68	7.91	37.36	34.1%	15%

**Notes.**

a Any foraging that was classified as occurring on land was assumed to be travelling.

The most energetically expensive behaviour was foraging, making up over a third of the DEE (Table 3). Resting on land made up ∼15% of the overall energetic budget though this was the largest part of the activity budget (∼45%). Fur seals spent little time resting at sea (∼10%) and this behaviour represented ∼9% of the overall energetic budget. The least costly activities were underwater grooming (1.4%) and resting (1.5%) which were likely to be mistaken behaviour classification. Grooming only made up one seventh of DEE, less than resting, and most grooming activity was at the surface (12%). Travelling and foraging made up two-thirds of the energetic budget, though they only represent roughly a third of the activity budget.

**Figure 6:**
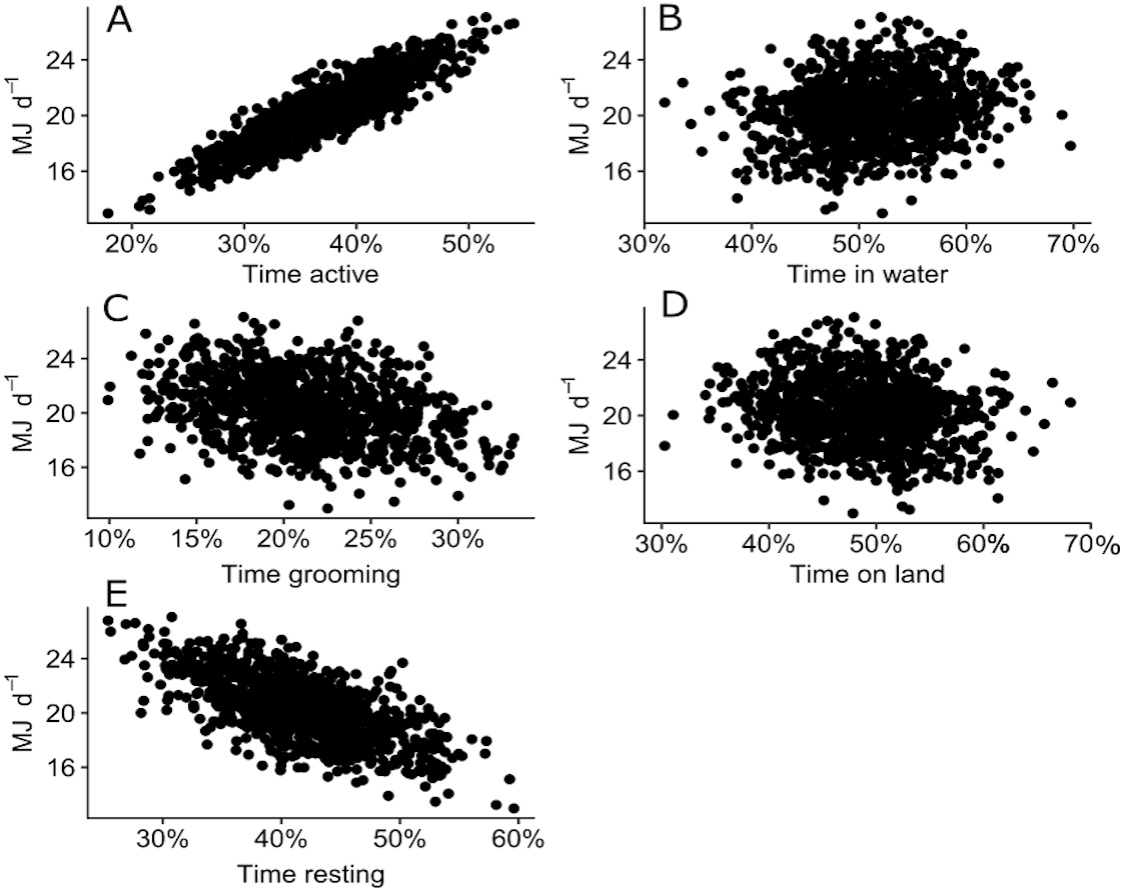
Plot of 500 simulated points of total DEE for wild juvenile Australian fur seals against percentage of time spent: (A) active (travelling and foraging); (B) grooming; (C) resting; (D) in water; (E) on land.

The sensitivity analysis revealed that the proportion of time spent in different behaviours (active, grooming or resting) and locations (water or land) altered the expected DEE for a juvenile Australian fur seal. DEE increased with more time spent active where DEE increased up to 27 MJ d^−1^ when a seal was active more that 50% of the time (Fig. 6A). DEE decreased to 13 MJ d^−1^ as seals spent more time resting (Fig. 6C). There was no clear relationship between the time spent grooming (Fig. 6B) or time spent on land or in water (Figs. 6D–6E) and DEE.

## Discussion

### Behaviour segmenting with accelerometers and machine learning

Supervised machine learning models trained with accelerometry data from captive animals reliably and accurately classified all four behaviour categories tested: foraging, grooming, resting and travelling. We expected that higher resolution data would enable the models to perform better at distinguishing the different behaviour types (Halsey et al., 2009), but were unable to compare this directly as we had too few long-duration behaviours recorded in water at high resolutions (>7 s). Despite this limitation, low resolution sampling produced very high out-of-sample (i.e., test) validation accuracies over a range of epoch sizes (72–90%; Table 2). The sample window size (epoch) influenced the result, with longer epochs tending to produce greater accuracies. The overall value of the summary statistic and its variation would be lower for long duration behaviours, i.e., low energy, repetitive behaviours are easier to distinguish (Diosdado et al., 2015). Shorter epochs are more likely to pick up irregular movements of the animal that arise from short duration, high energy activities (such as burst attacks on prey), that were rarely seen in the captive experiments (Bom et al., 2014). Instead, foraging was defined by handling of dead prey and actively searching the bottom of the pool for food hidden in the substrate (Ladds et al., 2017b). From this definition, foraging became the most difficult behaviour category for the model to classify.

The trained model based on captive animals predicted the behaviour of the wild fur seals, with minimal anomalies. Overall, the activity budgets matched expectations of wild fur seals (Battaile et al., 2015), where they spent most time resting (∼45%), and the rest of their time was split evenly among other activities—grooming (∼22%), foraging (∼20%) and travelling (∼12%). The model classified some behaviours incorrectly, primarily from two events—classifying the descent of the dive as foraging, when it was more likely travelling, and from identifying bouts of foraging during long bouts of surface swimming transiting back to the colony, when the fur seals were more likely grooming or simply changing direction. Explicitly defining some behaviours from accelerometers for other species has also been challenging. For example, using supervised machine learning models the foraging behaviour of plovers could not be classified (Bom et al., 2014), nor grooming of pumas (Wang et al., 2015). The total time that fur seals spent foraging and travelling is likely accurate because foraging and travelling were most often confused, thus combining the behaviours reduces the overall error, and is considered sufficient to summarise their activity budget. One major limitation of this study was the use of dead prey to induce foraging, so future studies should use live prey to help refine and improve the accuracy of models.

### Time-energy budgets

Juvenile fur seals had an average DEE that was 4.2 times the predicted BMR, which was about the same as estimated mass-specific energy expenditure measured from adult female Antarctic fur seals and northern fur seals (4.7 times BMR; Jeanniard-du Dot et al., 2016) and slightly lower than adult female Californian sea lions (*Zalophus californianus californianus*) (5.2 times BMR; Ponganis et al., 1997). DEE did not differ for sex or for season (winter vs. summer) allowing us to pool the data. Sample size was too small and without adequate replication for further division. Even so, sex differences were not expected for juveniles, as any differences in diving abilities (Fowler et al., 2006), physiological parameters (Burns, Clark & Richmond, 2004) or survival (Beauplet et al., 2005) have been attributed to age or size rather than sex (Weise & Costa, 2007).

#### Grooming

Juvenile fur seals in this study spent approximately 25% of their time at-sea grooming, compared to northern fur seals that spend around ∼30% of their time at sea rolling at the surface and another 9% in other grooming activities (Battaile et al., 2015). Fur seals groom for general body maintenance and this can offset some of the costs of thermoregulation (Iwata et al., 2013; Liwanag, 2010). Through rubbing the fur, fur seals encourage small air-bubbles to be accumulate between their layers of fur, providing further insulation. This also helps maintain positive buoyancy which in turn saves energy while diving (discussed below; Fish et al., 2002) and increases metabolic rate in cold water (Liwanag, 2010). Fur becomes compressed while diving, reducing its effectiveness to provide insulation, warmth and buoyancy (Fish et al., 2002), and to counteract this fur seals roll at the surface while rubbing their body with their fore flippers in order to trap air bubbles into their pelage (Liwanag, 2010). Indeed, our models identified many cases of grooming following a dive (e.g., Fig. 4).

Other thermoregulatory behaviours are sailing and jughandling which allows heat to escape their hairless flippers in warm water, or to avoid heat loss in cold water (Bartholomew & Wilke, 1956). Due to the sedentary style of this behaviour, the total energetic cost of daily grooming was not different from resting, despite having up to twice the energy demand (Liwanag, 2010). Fur seals appear to allow for its increased energetic cost by resting more often (Table 3). General body maintenance, such as rubbing whiskers, can occur after consuming large prey items. Wild polar bears (*Ursus maritimus*) have been observed to clean regularly while consuming prey where they pause eating at regular intervals to rinse and lick their fore paws and face (Stirling, 1974). Fur seals also spent significant time grooming on land (∼15% of all land activity) using their flippers and occasionally their teeth to maintain their fur, further indicating the importance of this behaviour.

#### Resting

Juvenile fur seals spent around half of their time resting, which contributed around ∼14% to their overall energetic budget. Due to the large cost of travelling and foraging, fur seals must use long haul-out periods to rest and recuperate. This is particularly true of juveniles who have an additional cost of growth, and use this time for reintegrating tissue and laying down fat (Kirsch, Iverson & Bowen, 2000). Juvenile Australian fur seals spent on average 72% of their time on land resting, which was ∼16% of their overall activity budget. During long periods ashore, fur seals generally remain motionless for energy conservation while fasting. For example, over the breeding season, adult male northern and subantarctic fur seals spent >90% of observed time (during the day only) motionless, either sitting or lying (Stirling, 1971).

The fur seals in this study spent ∼12% of their time at-sea resting, similar to Northern fur seals and Antarctic fur seals (*Arctocephalus gazella*) (Battaile et al., 2015), with ∼2% of this time underwater. Some phocid seals rest underwater (Mitani et al., 2009), but it is highly unlikely that these seals did so as Australian fur seal dives were generally only a few minutes, and their trip durations relatively short (Maresh et al., 2015). Instead, the behaviour classified as resting underwater may be explained by the model classifying the ascent part of the dive as resting. Long periods of gliding on the ascent part of the dive, likely results from the fur seals being positively buoyant. During underwater glides, metabolic rate is at or lower than RMR (Fahlman et al., 2008), which conserves their on-board oxygen stores (Ponganis, Meir & Williams, 2011; Williams et al., 2004). Therefore, classifying this part of the dive as resting, and thus having a lower metabolic rate associated, actually strengthens the validity of the models.

#### Active behaviours (Foraging and Travelling)

Derivation of our energetic budget distinguishes between two sedentary behaviours (resting and grooming) and two active behaviours (foraging and travelling). While there has been a proposition that accelerometers can be used to measure energetics from active behaviours (Jeanniard-du Dot et al., 2016), these relationships are confounded by time (Halsey, 2017). To account for this, we estimated active energy expenditure as a function of time spent active at sea. This approach assumed that fur seals were postabsorptive at sea and postprandial on land and the cost of foraging and travelling were equivalent. These assumptions are supported by evidence that seals partially delay digestion while diving (Rosen & Trites, 1997). The cost of foraging and travelling in this study could not be separated because the model sometimes confused the behaviours. Regardless, the two behaviours are inextricably linked due to the common movement of the behaviours (Ladds et al., 2017b), and the energetic cost would likely be similar.

Estimating the energetic cost of locomotion on land was difficult because this has not been measured for otariids. Movement on land is likely far more costly than in water because seal morphology has adapted them for efficiency in the ocean (Beentjes, 1990), a hypothesis with experimental evidence from the platypus and the water rat (Fish et al., 2001). Therefore, the cost of travelling on land was assumed to be twice as costly for fur seals as swimming in water. As a result, the average EE of activity on land was 1.51 MJ d^−1^, or ∼5% of the overall energetic budget. Given the assumed high cost of travelling on land and that travelling on land represented only ∼4% of the overall activity budget, juvenile fur seals likely minimise the time spent active on land to save energy for foraging.

Juvenile Australian fur seals spent around half of their time in water (36–69%). During the breeding season, adult female Australian fur seals spend around 75% of their time in the water (Arnould & Hindell, 2001), while pups are only in the water for around 29% of their time (Spence-Bailey, Verrier & Arnould, 2007). Of the time juveniles are in the water, approximately 56% (35–62%) is spent foraging and travelling, which contributes to most (∼75%) of their DEE (Fig. 4, Table 3). The large cost of travelling and foraging is predominantly from the mechanical power of flipper strokes during swimming, rather than diving, which can be offset using a range of behavioural compensatory techniques that lower metabolic rate (Davis & Williams, 2012).

## Conclusions

We constructed time-energy budgets for wild fur seals across multiple foraging trips using accelerometers recording at a low resolution (1 Hz), validating the activity budgets from experiments with captive surrogates. Sensitivity analyses revealed that the average DEE for a 50 kg wild juvenile Australian fur seal over multiple foraging trips was between 18 and 25 MJ d^−1^ which equated to 1.9 to 6.4 times Kleiber’s (1975) prediction for the BMR for similarly sized terrestrial mammals. This was within than the field metabolic rate (FMR) range of 3.3 to 6.7 times Kleiber reported for adult female otariids in other studies (Costa, Croxall & Duck, 1989; Fowler et al., 2007; Jeanniard-du Dot et al., 2016).

An important finding from this study is that time-energy budgets were able to be created from low resolution (1 Hz) accelerometry with very high accuracy (90%). Previous studies interpreting the foraging behaviour (Battaile et al., 2015) or energy expenditure (Jeanniard-du Dot et al., 2016) of wild fur seals have used high (>20 Hz) resolution data, at a cost of space and battery power from the device. Through validating low resolution accelerometry, this study will advance the use of accelerometers in the field as remote uploads are more feasible with less data, and battery life can be prolonged. Using low resolution data also significantly decreases the computational time and power required for analysis. Finally, as the drive towards smaller tags continue, using low resolution settings supports the use of smaller tags, without restricting the time with which they can be deployed.

While there is potential to fine-tune the model presented here to estimate a detailed time-energy budget on a minute or hourly basis, the current methodology provides a validated and representative estimate of daily time-energy budgets for wild fur seals. Through pairing estimates of energy expenditure with behaviour this study demonstrates the potential to understand not only how fur seals expend energy, but also where and how behavioural compensations are made to retain constant energy expenditure over short (a dive) and long (season) time period.

##  Supplemental Information

10.7717/peerj.5814/supp-1 Table S1 Summary statistics of daily energy expenditure (DEE MJ *d*^−1^) and dive trip details for nine juvenile Australian fur seals Fur seal details and average, standard deviation, minimum and maximum DEE for the length of deployment.

10.7717/peerj.5814/supp-2 Figure S1 An example of 12 hours of diving from a 40 kg juvenile Australian fur seal Panels show VeDBA, raw acceleration, location (underwater or surface), behaviour category (travelling, resting, foraging, grooming) and depth.

## Additional Information and Declarations

### Field Study Permissions

The following information was supplied relating to field study approvals (i.e., approving body and any reference numbers): This study was conducted under permits from Macquarie University ethics committee (ARA-2012_064) and Taronga ethics committee (4c/10/13).

### Data Availability

The following information was supplied regarding data availability: GitHub: https://github.com/MoniqueLadds/Time-energy_budgets_from_accelerometers.git.
