# Supplementary material for: Using accelerometers to develop time-energy budgets of wild fur seals from captive surrogates
Source: PeerJ. 2018 Oct 26;6:e5814. doi: 10.7717/peerj.5814 (PMC6204822; doi:10.7717/peerj.5814)
Supplement: Table S1 — Fur seal details and average, standard deviation, minimum and maximum DEE for the length of deployment. [file peerj-06-5814-s001.docx]

Summary statistics of daily energy expenditure (DEE MJ d^-1^) and dive trip details for nine juvenile Australian fur seals.

Fur seal details and average, standard deviation, minimum and maximum DEE for the length of deployment.

| ID | Sex | Mass (kg)  Length (cm) | Number of trips | Av. trip duration (days) (SD) | Number of dives | Av. max. dive depth (m) (SD) | Av. dive duration (s) (SD) | Max depth (m) | Av. DEE  (MJ d^-1^) (SD) | Range DEE | Av. Kleiber multiple | Days |
| --- | --- | --- | --- | --- | --- | --- | --- | --- | --- | --- | --- | --- |
| Winter |  |  |  |  |  |  |  |  |  |  |  |  |
| A09804 | Male | 41  130 | 2 | 5.8 (4.6) | 2907 | 40.3 (31.8) | 114.8 (85.6) | 85 | 21.2 (10.0) | 12.3-37.1 | 4.5 | 8 |
| A09867 | Female | 43  139 | 6 | 1.6 (0.4) | 3113 | 33.2 (8.6) | 128.6 (31.3) | 63 | 24.7 (7.8) | 12.0-37.3 | 5.0 | 16 |
| A09869 | Male | 45  133 | 7 | 1.1 (0.7) | 2158 | 51.4 (29.6) | 121.9 (59.1) | 81 | 25.2 (8.6) | 11.4-37.0 | 5.0 | 14 |
| Summer |  |  |  |  |  |  |  |  |  |  |  |  |
| A09844 | Female | 35  107 | 14 | 0.3 (0.2) | 1071 | 29.7 (21.3) | 121.9 (79.4) | 62 | 22.6 (4.5) | 17.2-33.0 | 5.4 | 19 |
| A09864 | Female | 30  110 | 4 | 1.2 (0.9) | 1261 | 73.2 (23.0) | 140.3 (39.9) | 105 | 27.8 (6.7) | 18.0-35.6 | 7.4 | 10 |
| A10281 | Female | 42  119 | 12 | 0.8 (0.8) | 2499 | 34.9 (9.5) | 122.6 (38.3) | 54 | 26.2 (7.8) | 15.5-37.7 | 5.4 | 22 |
| A10282 | Female | 30  107 | 6 | 0.8 (1.0) | 1461 | 39.0 (8.9) | 173.2 (70.9) | 50 | 25.2 (6.5) | 14.9-33.8 | 6.7 | 19 |
| A10283 | Male | 34  110 | 45 | 0.1 (0.1) | 1552 | 10.2 (7.8) | 49.6 (36.2) | 44 | 25.6 (3.0) | 18.8-31.8 | 6.2 | 19 |
| A10284 | Female | 35  108 | 21 | 0.2 (0.2) | 1277 | 23.1 (12.7) | 90.9 (47.7) | 46 | 30.4 (6.8) | 15.8-38.9 | 7.2 | 18 |
