# Supplementary figures and images for: Using accelerometers to develop time-energy budgets of wild fur seals from captive surrogates
Source: PeerJ. 2018 Oct 26;6:e5814. doi: 10.7717/peerj.5814 (PMC6204822; doi:10.7717/peerj.5814)

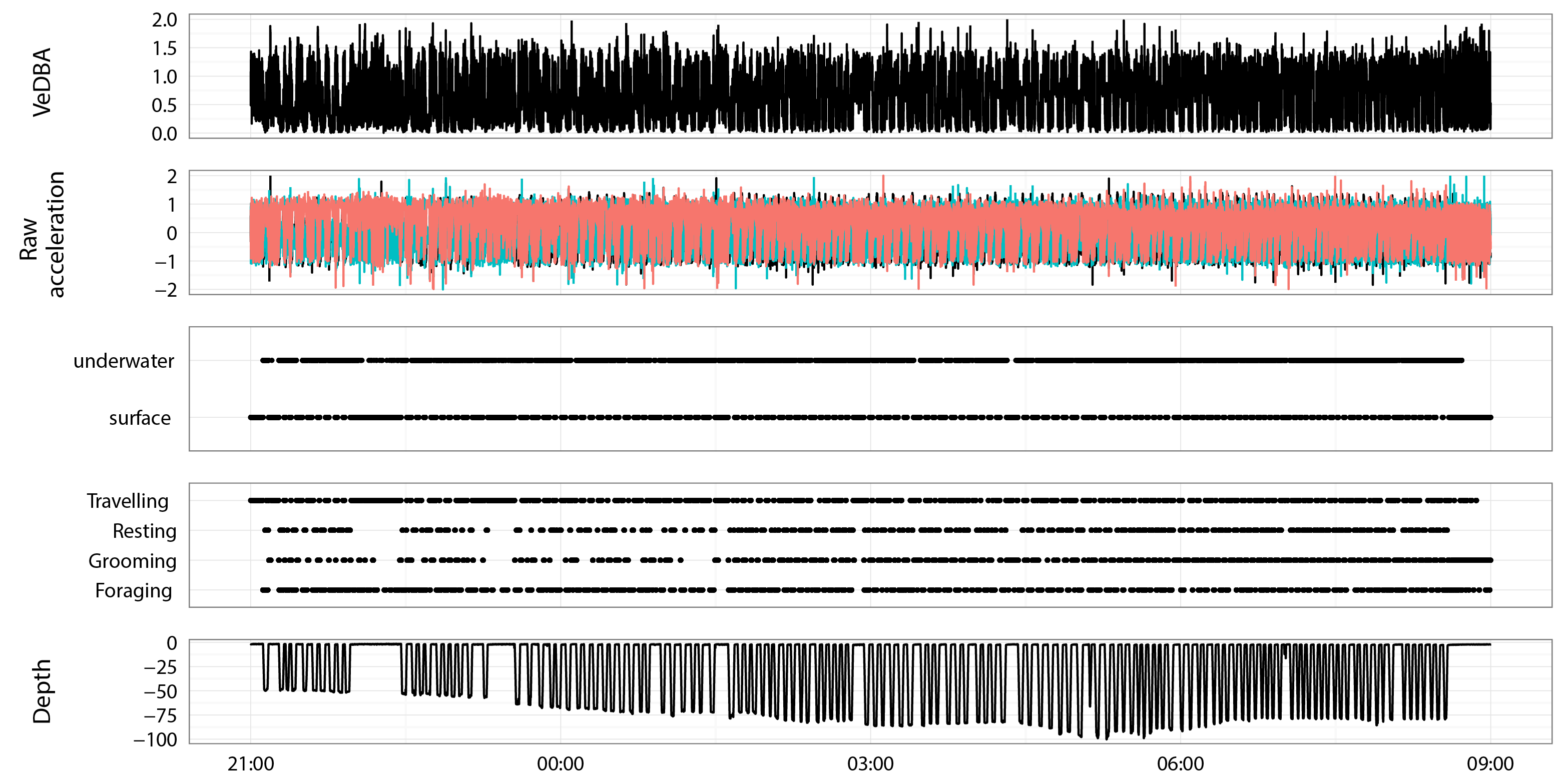

Supplement: Figure S1 — Panels show VeDBA, raw acceleration, location (underwater or surface), behaviour category (travelling, resting, foraging, grooming) and depth. [file peerj-06-5814-s002.png]
